# Impact of Polyploidy Induction for Salinity Stress Mitigation in Soybean (*Glycine max* L. Merrill)

**DOI:** 10.3390/plants12061356

**Published:** 2023-03-17

**Authors:** Phetole Mangena

**Affiliations:** Department of Biodiversity, School of Molecular and Life Sciences, Faculty of Science and Agriculture, University of Limpopo, Sovenga, Private Bag X1106, Polokwane 0727, South Africa; phetole.mangena@ul.ac.za; Tel.: +27-(0)15-268-4715

**Keywords:** biochemical traits, colchicine, *Glycine max*, morphology, oryzalin, polyploidy, salinity stress, soybean

## Abstract

Polyploidy induction is recognized as one of the major evolutionary processes leading to remarkable morphological, physiological, and genetic variations in plants. Soybean (*Glycine max* L.), also known as soja bean or soya bean, is an annual leguminous crop of the pea family (Fabaceae) that shares a paleopolypoidy history, dating back to approximately 56.5 million years ago with other leguminous crops such as cowpea and other *Glycine* specific polyploids. This crop has been documented as one of the polyploid complex species among legumes whose gene evolution and resultant adaptive growth characteristics following induced polyploidization has not been fully explored. Furthermore, no successfully established in vivo or in vitro based polyploidy induction protocols have been reported to date, particularly, with the intention to develop mutant plants showing strong resistance to abiotic salinity stress. This review, therefore, describes the role of synthetic polyploid plant production in soybean for the mitigation of high soil salt stress levels and how this evolving approach could be used to further enhance the nutritional, pharmaceutical and economic industrial value of soybeans. This review also addresses the challenges involved during the polyploidization process.

## 1. Introduction

**Figure 1:**
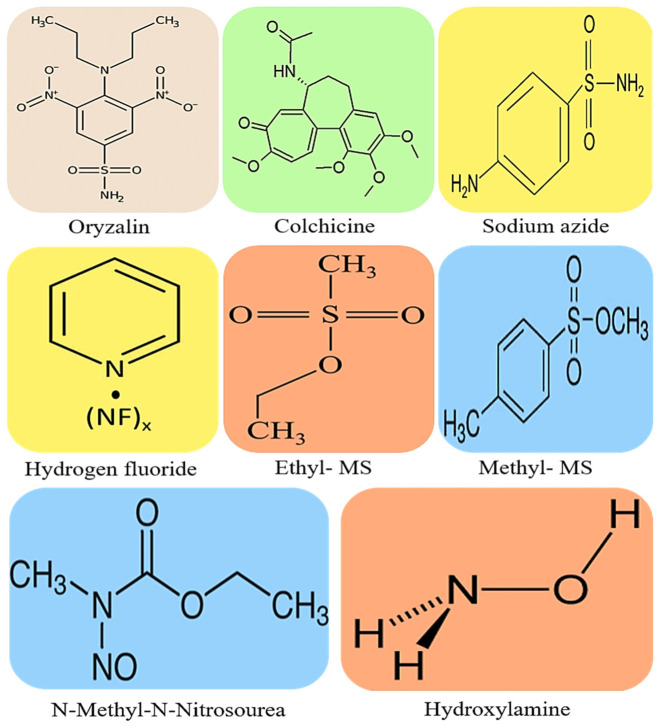
Chemical structure depictions of the most commonly used compounds for mutagenic crop improvement against biotic and abiotic stresses: oryzalin with a molecular weight (MW) of 346.36 g/mol, colchicine with a MW of 399.437 g/mol, sodium azide with a MW of 65.0099 g/mol, hydrogen fluoride with a MW of 20.0064 g/mol, ethyl methanesulfonate (Ethyl-MS) with a MW of 124.16 g/mol, methyl methanesulfonate (methyl-MS) with a MW of 110.13 g/mol, N-Methyl-N-Nitrosourea with a MW of 103.08 g/mol, and hydroxylamine with a MW of 33.03 mg/mol.

Polyploidy induction is recognized as one of the major evolutionary processes that leads to a variety of remarkable morphological, physiological, and genetic variations in plants [1,2,3]. In Fabaceous crops such as soybean, also known as soja bean or soya bean, Kim et al. [1] reported a paleopolypoidy history that dates back to approximately 56.5 million years ago, which is similar to that of common bean and other *Glycine* specific polyploids. This history of evolution and diversification of plant species also drove soybean domestication and the improvement of its agronomic as well as nutritional characteristics [4]. Both soybean’s evolutionary path and domestication led this crop to become one of the current major sources of dietary carbohydrates, fibre, minerals, oil, proteins and vitamins for human and animal consumption [5]. Generally, plant polyploidization consists of the multiplication of a complete set of chromosomes that co-exist within a single cell nucleus. Progenies, subsequently leading to the formation of diversified species’ mutant varieties [6], can stably inherit these newly formed genomic features. In leguminous plants and likewise in non-leguminous species such as rice, wheat, tomato, cutleaf groundcherry etc., polyploidization has been widely tested using chemicals such as colchicine [7], epoxomicin [8], sodium azide [9], oryzalin [10], nitroxide [11] and ethyl methanesulfonate [12], some of which are illustrated below in Figure 1. These chemical compounds cause numerous genetic mutations that result in significant changes in the plant’s nuclear and proteome systems. Although the induced mutations can cause genetic defects and undesirable deformities that are easily identified through phenotypic evaluations, the changes incurred also serve as an alternative means of achieving genetic variations which contribute to the much-needed species diversity. Mastuti et al. [13] reported variations in growth response characteristics of the different types of explants treated with 0.1% colchicine solution for 0, 24, 48 and 72 h in *Physalis angulate*. In specifically targeting the proteasome using epoxomicin, van der Hoorn et al. [14] demonstrated unexpected changes in *Arabidopsis*’ protein activities during defense and stress responses, even though epoxomicin has been widely reported mainly as an antitumoral natural product inhibiting primarily chymotrypsin-like activity [15]. Mutagenesis studies in *Saccharomyces cerevisiae* and other organisms, in combination with proteomics, revealed that this chemical indirectly induces irreversible mutations.

This chemical influences proteolytic systems in the cytosol and nucleus to alter cell growth and gene expression regulations in living cells [16,17,18,19]. However, nitroxide regulates plant morphogenesis and development through post-translational protein modifications, calcium pump regulation, hormonal regulations, and reductions in reactive oxygen species (ROS) activity [18]. Meanwhile, other studies show that oryzalin induced polyploidy in plants by disrupting microtubule formations, similar to the preventative anisotropic growth effects of colchicine [20]. Interestingly, for proper plant growth, vegetative development and reproduction under high salt concentrations, mutagenic chemicals could also be used to enhance morpho-physiological and biochemical adaptive response mechanisms of plants. The ability of plants to respond and survive under salinity stress remains a priority as this abiotic condition essentially reduces cell water potential causing dehydration and ion cytotoxicity. This stress, resulting from the combined effects of over-irrigation and poor soil drainage, disrupts soybean growth, as in many other plants, by hindering seed germination, seedling development, flowering and fruiting [11,20,21,22,23].

This review, therefore, describes the role of synthetic polyploid plant production in soybean for the mitigation of high soil salt stress levels, briefly discusses how this evolving approach could be used to further enhance the nutritional, pharmaceutical and economic value of this and other leguminous crops and addresses the challenges involved in the polyploidization process under both in vivo and in vitro conditions.

## 2. Genetic Architecture and Response to Salinity Stress in Soybean

**Figure 2:**
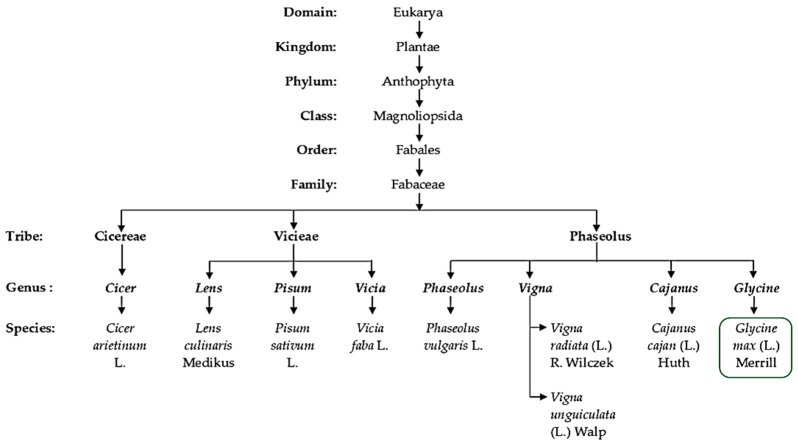
A summary of a taxonomic classification scheme highlighting the position of soybean (*Glycine max* L.) and other selected grain leguminous crops, as well as their botanical names (genus and species).

As a partial tetraploid, soybean is a legume belonging to the Fabales, which comprise more than 20,055 species, distributed within about 754 genera and making up nearly 10% of the eudicots [24,25]. Legumes experienced whole genome duplication after the Cretaceous–Paleogene (K–Pg) boundary (KPB) mass extension [25]. Especially, with allopolyploidy events giving rise to the ancestral species, *Glycine soja* is proposed to be a wild progenitor of *Glycine max* L. (soybean) [26]. Undoubtedly, species found within the Eurosid 1 angiosperm group constitute many of the most economically and agronomically important leguminous plants (Table 1 and Figure 2), in particular, species of the tribes *Vicieae*, *Cicereae*, *Dalbergieae*, *Genisteae*, *Indigofereae* and *Phaseoleae* [27,28]. Species from these selected families are used as food crops, directly or indirectly, in the form of ripe-mature or unripe-immature pods, as well as mature and immature dry seeds. Figure 2 shows the taxonomic classification of cultivated leguminous species used as grain and forage crops for human and animal consumption. Among them, soybean falls within the *Phaseoleae* tribe with prominent and widely domesticated species such as pigeon pea, dry bean, black gram, mung bean and cowpea, as presented with their scientific names in Table 1. However, a species-comprehensive phylogenetic illustration (Figure 2) is important for capturing the full species diversity and taxa relationships for most cultivated grain legumes belonging to all natural tribes, especially the *Vicieae* and *Phaseoleae*. Both tribes consist of species exhibiting similar phylogenetic characters needed to overcome food insecurity and limitations such as inefficient nodulation, and resistance to biotic and abiotic stress constraints. Furthermore, the species within these tribes belong to a super clade called eudicot, which clearly demonstrates diversity in morphology, physiology, ecology and anatomical/structural support mechanisms in response to stresses such as drought and salinity [29]. Like many of these plants, soybean evolutionarily contains inherent morpho-physiological and biochemical mechanisms that permit it to thrive under high salt stress environments. Taxonomically similar to other species within the tribe, which is traditionally regarded as the clade of unnatural taxon (Figure 2), with mainly edible species [30], this crop exhibits growth features required to cope with significant levels of abiotic stress.

Among the complex and elegant strategies to overcome abiotic salt stress, soybeans convert stress signals to alter gene expression in order to activate mechanisms of acclimation and tolerance. The first salt stress-sensing mechanism triggers a downstream response comprising multiple signal transduction pathways. These pathways involve activation of transcriptional regulators, ROS signaling and accumulation of secondary metabolites such as hormones, among others [22]. This signaling, in turn, regulates metabolic and gene expression reprogramming that bring about cellular stability under salinity conditions. According to Zhu [31], stress signaling also controls protein expression, which serves as a critical mechanism for ion and water homeostasis in the cells. As a result, salinity stress-mediated transductions that emerge from these pathways in turn activate or suppress various networks within soybean that may either allow growth to continue under stress conditions or enable the plant to buffer its growth and reproduction until more favourable conditions are achieved. Generally, most the literature on plant polyploidy and salt stress does not provide measurements of how soybean polyploids, compared with their diploid counterparts, uniquely mediate cytotoxicity or osmotic stress induced by salinity stress, but rather reports on the changes in morpho-physiological and biochemical traits. Manzoor et al. [2] indicated that polyploidised plants developed from colchicine treated seeds showed increased adaptation to salt stress by efficiently regulating water use by varying osmotic potential of the leaf sap. Plant polyploids showed better adaptive character than diploid plants in regulating cation and ion toxicity that restrict enzyme activity and nucleic metabolisms. As this information on adaptive changes to salt stress by polyploidised soybean plants remains elusive, the question of how soybean plants modify their morphological, physiological and molecular architecture in order to adapt to adverse environmental conditions such as salinity, drought and pathogens remains critical for agricultural productivity and sustainability.

## 3. Improvement of Salt Stress Tolerance via Polyploidy Induction in Plants

**Figure 3:**
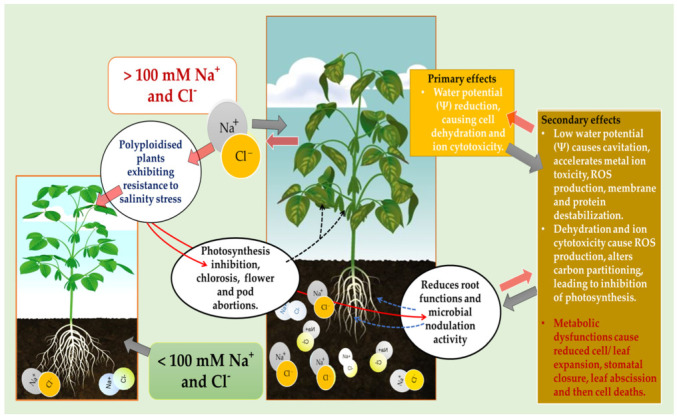
Potential effects of salinity on the growth and development of polyploidized and diploid soybean plants.

The yield of soybean is significantly decreased by salinity stress, especially by the negative influence on the vegetative growth stages of this crop, as represented in Figure 3. Salinity is predicted to affect at least 50% of cultivated land worldwide by the year 2050 [32]. This stress causes primary negative effects by reducing cell water potential, causing dehydration and ion cytotoxicity, and secondary effects leading to ROS production, membrane destabilization, protein degradation and inhibition of photosynthesis (Figure 2). As previously emphasized, salinity inhibits growth and development of the whole plant by causing ROS accumulation, and water and ionic imbalances in plant cells [21,32,33]. The Foyer–Halliwell–Asada pathway, also known as the glutathione-ascorbate cycle, that detoxifies ROS is one major mechanism used by the genetically enhanced polyploids to tolerate and survive salinity stress [34]. The ascorbate–glutathione or glutathione–ascorbate cycle eliminates ROS through the activity of ascorbate peroxidase, monodehydroascorbate reductase, dehydroascorbate reductase and glutathione reductase. These enzymes play a vital role in detoxifying ROS effects, as indicated in Figure 3, especially during abiotic stress where the harmful oxygen free radical production is increasing, even surpassing the antioxidant defense capacity of diploid plants compared with the polyploidised ones [34,35].

Apart from ROS detoxification, plants also use compartmentalization or exportation of ions to different internal/external structures to achieve suitable osmotic adjustments. In this way, plants are generally able to regulate Na^+^/Cl^−^ ion uptake and carry out long-distance transport and intracellular compartmentalization in the vacuole and other specialized tissues to avoid excessive salt accumulation and cell damage by Na^+^/Cl^−^ influx [36]. For example, halophytes such as *Aeluropus*, *Astriplex*, *Cakila*, *Mesembryanthemum*, *Imperata*, *Salicornia*, *Suaeda* and *Trellungiella* have greater abilities to coordinate the distribution of salts through unique structures, such as bladders, salt glands and succulence, to excrete salts from their internal organs [36,37]. Similar observations were made in *Betula papyrifera* polyploids. These plants demonstrated enhanced accumulation of osmolytes under water-deficit and salt stress in different polyploid lines, with similar processes observed in polyploids of citrus and tea species [31,34,36,37,38].

### 3.1. Vegetative Growth Characteristics for Salinity Tolerance

Induced polyploidization as a tool for improving crop traits was discovered in 1907, and was thought to be responsible for heritability in genomic characteristics [2]. It was then later demonstrated that different ploidy levels caused different effects on the morphology and physiology of plants. As reported earlier by Alam et al. [38], tetraploid and triploid *Camellia sinensis* mutant plants showed more vigour and leaf hardness due to increased sizes of cortical and mesophyll cells, which increased eight-fold compared with six-fold in diploid cells. However, evidence of the effects of synthetic polyploidy application in legumes, particularly, in soybean is very limited. A few detailed and insightful scholarly works that are available about polyploidy at all levels clearly show that this phenomenon induces changes in plant phenotypes via the altered genome, also influencing interactions with abiotic and biotic environmental stress factors [39]. Forrester and Ashman [39] reported that polyploidy directly increased the quantity and quality of rhizobia symbionts found in legumes such as *Glycine wightii*, *Medicago sativa*, *Stylosanthes hamata* and several *Trifolium* spp., resulting in enhanced nitrogen (N) fixation due to larger quantities of nodules (8.8 to 119.8), improved nodule sizes (up to 3.0 mm) and higher root density (0.21 cm^−2^cm^−3^ to 1.38 cm^−2^cm^−3^). In cowpea, attempts have been made using this tool to improve primitive and long-existing characteristics such as small seed size, hairiness, and exined pollen grain surfaces [40].

*Vicia cracca*, another Fabaceae, was also used by Munzbergova [41] as a model taxon to reveal larger seed weight (>187.22 g/plant, *p*-value <0.001) and stomata (172.95 µm, *p*-value <0.001), which were influenced by colchicine application during polyploidization that was performed as described by Pavlikova et al. [42]. In addition, a larger number of morphological effects have been detected in non-leguminous but mostly ornamental plant species, such horticultural and ornamental plants with commercial value that include *Rhododendron fortunei* [43], *Trachyspermum ammi* L. [44], *Taraxacum kok-saghyz* [45], *Lilium regale* [46], *Chrysanthemum carinatum* L. [47], as well as other species that are not cited in this paper. In some of these species, such as the Chinese privet (*Ligustrum sinense* Lour.), higher frequencies of pure tetraploids (30.8%) at lower seedling mortality after treatment with 0.1 or 0.2% colchicine, resulted in reduced morphological changes (compact plants, shorter, with 20.1% fewer branches), but with 48.0% larger leaves and 71.8% higher fresh as well as dry weights [38]. However, in view of above-mentioned reports, artificially multiplying the chromosome complement in soybean to influence morphological changes and responses to abiotic salinity stress remains a daunting task. This is due to the formation of aneuploidy, as earlier reported by Palmer [48]. In most cases, aneuploidy leads to the lack of morphological variations among the expected polyploidy progenies, loss of pollen fertility, reduced number of seeds and plant growth retardation.

### 3.2. Enhanced Biochemical and Physiological Responses

Generally, polyploidization leading to changes in the number of chromosomes serves as a critical source of genetic variations required to improve desirable traits in many crop species. Changes in chromosome number affect the nuclear genome, which in turn influences enzyme production and activity, then finally affect the plant’s traits. The changes in the chromosome number also lead to quantifiable effects on the physiological and biochemical characteristics of the plant. Apart from the nuclear DNA content changes induced during polyploidization, Luo et al. [45] reported significant fluctuations in the concentrations of inulin (33.4 mg/g in tetraploid compared with 47.32 mg/g in diploid plants), sugar (27.45 mg/g in tetraploids compared with 33.48 mg/g in diploid plants) and resin (57.58 mg/g in tetraploid compared with 34.58 mg/g in diploid plants) in *Taraxacum kok-saghyz* seedlings developed by treating seeds with 0 to 0.5% colchicine for 12 to 96 h. Noori et al. [44] also reported two-and-a-half times more oil yield in colchicine induced tetraploid than diploid control plants using 0.025 to 0.5% for 6 to 48 h in ajowan (*Trachyspermum ammi* L.). Tetraploid plants produced 69.2% of thymol content in essential oil in contrast with those of diploid plants at 49.67%. In *R. fortunii*, polyploidised tetraploids and octoploids were found to contain significantly higher amounts of total chlorophyll, with 76.37 and 117.80 mg/g, respectively, compared with 41.19 mg/g in their diploid counterparts [43]. The application of colchicine as the most common procedure used for chromosome doubling, enhancing both biochemical and physiological parameters, has proved especially successful in monocot species, such as barley, maize and wheat, allowing an increased gene scope and expression, as reported by Rauf et al. [49]. The approach also reduces deleterious effects induced by genomic and epigenomic instability. Crops such as maize, barley and wheat showed increased leaf protein content, total soluble solids, sucrose and water-soluble carbohydrates following induced polyploidy germplasm [49,50,51]. Although polyploidy induction has been used to improve a number of biochemical parameters in many species, genome duplication has been induced at over 75% efficiency in over 120,000 species of grasses and legumes mainly used as forage crops [50,51]. Such improvements in the performance of plants can be attributed to the biochemical and physiological changes induced by the anti-tubulin effects of acetamide, oryzalin (Figure 1), colchicine (Figure 1), amiprophos-methyl and trifluralin [49,52].

## 4. Ploidy Stability and Molecular Profile for Salinity Stress Resistance in Soybean

As already defined, polyploidy is a natural phenomenon responsible for reduplication of sets of chromosomes in plant species occurring through autopolyploidy or allopolyploidy. However, as a result of genetic instability and sterility, many of these polyploid species require propagation through vegetative cloning. For instance, in banana, maximum vigour and quality are maintained generation after generation through vegetative propagation of plants with the triploid state. As reported by Lestari et al. [53], similarities in the number of banana tillers, brix percentage, fruit peel/mesocarp thickness and fruit length serve as indicators of genetic stability, wherein the genetic similarity coefficient ranged between 0.9 and 1.0. Nevertheless, under stressful conditions such as salinity stress, polyploidised plants are expected to demonstrate higher adaptive value or fitness (Figure 3) than their diploid counterparts. Furthermore, in the case of triploid bananas being sterile, the resultant fruit becomes seedless and more commercially acceptable. Other polyploid species include commercial cultivars of potato and sugarcane that are vegetatively propagated to maintain their genetic stability and integrity. The pollen and ovule development of these cultivars can be ignored, even if they are normal and viable, particularly in order to maintain good genetic stock. Therefore, in soybean, as in many other legumes of Fabales (Figure 2), polyploidy could be explored for the development of unique bean germplasm that would morphologically and physiologically contribute to improved yield under biotic and abiotic stress conditions. Additionally, this could also be based on the need to increase genetic diversity and improve the genetic base that may come with enhanced architectural and yield traits. Nevertheless, polyploidy is well tolerated in many crop species, with the majority of plants being descendants from polyploidized ancestors. Other long-term or short-term effects such as genomic stability, gene expression and genetic redundancy still need to be fully evaluated.

Wendel et al. [54] reported that polyploidization has both immediate as well as long-term genomic and transcriptomic consequences, which lead to different fitness, biased expression of homologs (gene duplicated by polyploidy) and loss of certain sequences from the duplicated genome. As reported by Kolar et al. [55], polyploidy leads to a unique form of genomic and phenotypic variation that influences crop species interactions with their environment. This process may result in high crop species fitness that is postulated to confer resistance to abiotic constraints such as salinity stress. In *Malus domestica* (apple), in the cultivars Hanfu and Gala, induced autotetraploid plants were found to respond better to salt stress (200 mmolL^−1^ NaCl) than diploid plants [56]. Similarly, tetraploids of *Carrizo citrange* seedlings were reported to be more tolerant to salt stress (40 mM NaCl for 20 days) [57] and *Citrus limonia* Osb. seedlings more adapted to nutrient deficiency for over 7 months than their diploid genotypes [58]. Although numerous studies already provide insights into polyploidy-induced mechanisms of salinity tolerance in citrus crops, better understanding and evidence of induced changes in the physiological functions, as well as gene expressions, are required in leguminous crops such as soybean. Less clear and still needing investigation is the magnitude of stress tolerance conferred by polyploidized species and how potential barriers such as genetic instability or redundancy can be overcome. Although polyploidy can be achieved in most crop species of angiosperm dicots, its potential to improve tolerance to environmental stresses still requires more investigation, and perhaps tolerance to heat, drought and salt stress will be found in legumes, including important horticultural species such as *Lycium ruthenicum* [59], *Asparagus officinalis* L. [60], *Ziziphus jujube* Mill. Var. spinosa [61] and *Malus prunifolia* [62].

## 5. Potential Undesirable Ploidy Effects

Although polyploidy remains well tolerated in many eukaryotic organisms, as well as in the majority of angiosperms descended from polyploid ancestors, as previously indicated [63], the changes in genome structure may lead to immediate unintended undesirable effects on the genotype, phenotype and fitness of individual plant species. Such changes may allow beneficial evolutionary transitions that were not previously possible to occur, but with emerging serious consequences. Polyploidization can stimulate further structural changes in genomes, leading to disrupted normal cell growth, metabolism and regulation of the mitotic or meiotic cell cycle. This has been particularly been observed in animal cells more than in other living organisms, including plants [55,63]. At an ecosystem level, Weiss-Schneeweiss et al. [64] highlighted several fitness disadvantages, such as stigma clogging, dispersal limitations and higher water-use efficiency of tetraploid plants. Moreover, this involves genome rearrangements, including repetitive DNA and locus silencing (nuclear dominance), interlocus recombination and complete or near-complete repeat replacement or redistribution [64], that may lead to considerable losses of chromosomes. In soybean, aneuploidy commonly occurs, which is primarily a trisomy comprising an extra complete chromosome (2n = 2x + 1 = 41) observed in asynaptic and desynaptic mutant progeny [65]. This cause of meiotic mutation often leads to low fitness and poor reproductive capacity of soybean mutant polyploids.

For instance, in animals, aneuploidy or any ploidy changes are typically fatal, with polyploids dying early during their fetal development [66]. However, the available data indicate that polyploidization contributes to evolutionary successes by increasing embryo survival, seed longevity and species richness of polyploids compared with non-polyploidized individuals within many genera in plants. Furthermore, polyploidy shapes the geographical range and distribution of species [67]. Taxonomically, phylogenetic data also reveal that the frequency of polyploids increases with increasing latitude or any forms of environmental extremes, such as high temperature, drought and salinity [68]. Nevertheless, the better colonizing capabilities of polyploids, especially under abovementioned stressful environmental conditions, affect polyploid and non-polyploid plants differently. These effects involve polyploidy response to stress, which also include salinity stress. Moreover, polyploidy, particularly the synthetic production of mutant plants through the use of chemicals such as colchicine, still has the potential to play a significant role in the physiology and development of plants through cellular, metabolic and genetic effects [69].

## 6. Future Prospects and Final Considerations

**Figure 4:**
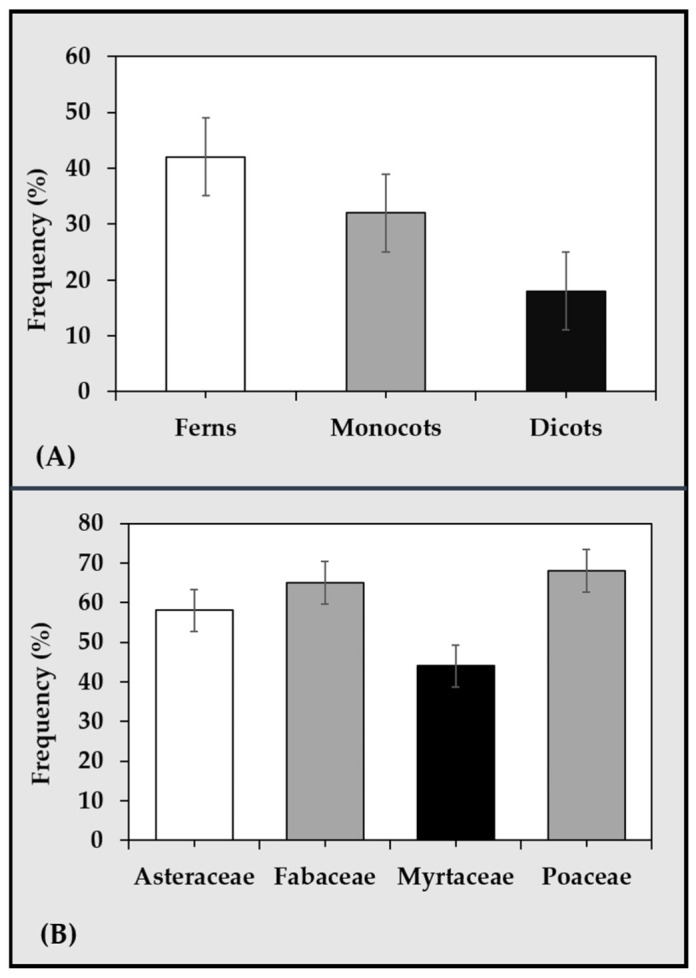
Estimates of the frequency (percentage) of natural polyploidization occurrences in vascular seed and seedless plants (**A**) [66] and among the major families of angiosperms comprising species used as food and feed crops (**B**) [71].

Van de Peer et al. [68] reported genome duplication in plants as a phenomenon that has reached a dead-end, particularly under stable conditions. The study postulated that, under natural conditions, changes in whole genome duplication predominantly coincided with the periods of major global climatic fluctuations. For instance, these variations overlapped with the Cretaceous–Paleogene boundary. This implies that the frequency of polyploidy formation in living organisms is driven primarily by external environmental stimuli such as stress, especially at a level of cataclysmic events [70]. The fact that ploidy number shows remarkable changes in response to environmental stress among plant species suggests that this can be further explored from an adaptation and mitigation point of view. Such predictable consequences of polyploidization resulting from the fusion or fission of chromosomes have been recorded over time, as shown by the readily available taxonomic data across groups of ferns and angiosperms. Natural polyploidization occurrences in plants are widespread and estimates are shown in Figure 4, together with the estimates of the proportions of polyploids found among some of the major families of angiosperms. This includes the Fabaceae family comprising soybeans and other leguminous crop species. As incidences of polyploidization are mapped according to taxonomic groups, phylogenetic position, habitat and phytogeographical region, the herbaceous leguminous species of Fabales, particularly of the Phaseolus tribe (including *Cajan*, *Glycine*, *Phaseolus* and *Vigna*) (Figure 3), exhibited higher basic chromosome numbers with considerable polyploidy of about 40%. This percentage includes species of the subfamily Caesalpinioideae, Mimosoideae and Papilionoideae [66,71].

Genomic studies have revealed that soybean developed from a tetraploid ancestor, and cytogenetics and molecular analysis showed that allopolyploidization played a key role in the speciation of this crop. Furthermore, more wild perennial species of the subgenus *Glycine* forming tetraploid and aneutetraploid ploidy complexes were also discovered [72]. Polyploids are a prevalent source and rich reservoir of desirable agronomic genes. Therefore, both naturally occurring and synthetic polyploids could be explored to unlock these valuable genes, which has so far not been successful. As such, this aneupolyploidy, as well as other genetic barriers, has caused the improvement of soybean to lag behind other economically important crops such as barley, faba bean, maize, tomato, rice and wheat. Wide crosses and dual polyploidization have been explored for yield improvement in rice. Cai et al. [73] earlier reported selective breeding of two polyploid lines, PMeS-1 and PMeS-2, using polyploid meiosis stability (PMeS) genes to breed japonica rice with a higher rate of seed set. Compared with monocot species such as barley, maize and rice, leguminous dicot polyploid lines of faba bean (*Vicia faba* L.) showed an unusually strong interaction between resistance genes and avirulence pathogen genes conferring resistance to *Uromyces viciae-fabae* [74]. This study demonstrated the significant role that polyploid lines may play in crops threatened by abiotic stresses and diseases. Such mutations can also be beneficially utilised to genetically improve varieties of soybeans, especially for enhancing adaptive properties of the crop plant against stresses such as drought and salinity.

## 7. Conclusions

The genetic base of soybean, particularly that of cultivars that are currently grown for both commercial and subsistence farming, is extremely narrow. This makes soybean a very poor reservoir for genes conferring tolerance to abiotic and biotic stress. However, the effects of these stresses are widely documented, especially the influence of salinity on soybean growth, development and yield. The potential for induced polyploidy in soybean against salinity stress remains less recognized and largely unexplored. This paper demonstrates without doubt that polyploidy can be used to confer resistance to different kinds of environmental stresses and can act as a reliable source of mutations to improve genetic variability in soybean. However, evidence is still required that may provide further insights into the mechanisms underlying the responses of polyploids to growth and stress-related phenomena, which might not differ greatly between polyploidised plants and their diploid counterparts. Under natural conditions, evolutionary changes in recalcitrant legumes such as soybean gradually take place due to aneuploidy or aneutetraploidy that has restricted genetic variability and wider geographical distribution for most of the *Glycine* species. In contrast, mutagenic chemicals such as those shown in Figure 1 can be used to successfully induce the desired genomic changes under controlled environmental conditions, also in a short space of time.

## Figures and Tables

**Table 1:** Species of legumes found within the Eurosid I angiosperm group (Fabidae) constituting some of the most economically important Fabale crops, globally [28].

Tribe	Species Name	Common Name
Cicereae	*Cicer arietinum*	chickpea
Dalbergieae	*Arachis hypogeae*	Peanut
Genisteae	*Lupinus luteus*	Yellow lupin
Indigofereae	*Cyamopsis tetragonoloba*	Guar
**Phaseoleae**	*Cajanus cajan*	Pigeon pea
	*Canavalia ensiformis*	Horse bean
	***Glycine max***	**Soybean**
	*Mucuna pruriens*	Velvet bean
	*Phaseolus lanatus*	Butter bean
	*Phaseolus acutifolius*	Tepary bean
	*Phaseolus vulgaris*	Dry bean
	*Vigna radiata*	Mung bean
	*Vigna mungo*	Black gram
	*Vigna umbellate*	Rice bean
	*Vigna unguiculata*	Cowpea
Vicieae	*Lens culinaris*	Lentil
	*Pisum sativum*	Pea
	*Vicia faba*	Broad bean

## Data Availability

Data is contained within the manuscript.
